# Community-Based Screening, Brief Intervention, and Referral for Treatment for Unhealthy Tobacco Use: Single Arm Study Experience and Implementation Success in Rural and Semi-Rural Settings, South-West Nigeria

**DOI:** 10.3389/fpsyt.2016.00134

**Published:** 2016-08-02

**Authors:** Victor Olufolahan Lasebikan, Bolanle Adeyemi Ola

**Affiliations:** ^1^Department of Psychiatry, College of Medicine, University of Ibadan, Ibadan, Nigeria; ^2^Department of Behavioral Sciences, Lagos State University, Lagos, Nigeria

**Keywords:** screening, brief intervention, community, tobacco, smoking

## Abstract

**Objective:**

To determine whether screening, brief intervention, and referral for treatment can reduce the prevalence of tobacco use in rural and semi-rural settings.

**Method:**

*Design and participants*: A non-randomized clinical trial with assessments at baseline and post-intervention assessments at 3 and 6 months was conducted in a rural and semi-rural district in South-West of Nigeria. A representative sample of 1203 persons consented to the study and had alcohol, smoking, and substance involvement screening test (ASSIST) administered to them by trained community health-care extension workers between October 2010 and April 2011. Follow-up participation was more than 99% at all points. *Intervention*: Participants received a single ASSIST-linked brief intervention (BI) and referral for treatment (RT) at entry, and a booster ASSIST BI and RT at 3 months. *Main outcomes and measures*: The primary outcome was self-reported scores on ASSIST.

**Results:**

At baseline, out of 1203 respondents, lifetime prevalence and current prevalence of any tobacco products were 405 (33.7%) and 248 (20.6%), respectively. Of the current users, on the ASSIST, 79 (31.9%) scored 0–3 (low health risk), 130 (52.4%) scored 4–26 (moderate risk), and 39 (15.7%) scored 27+ (high risk). At 3 months, out of 1199 respondents, prevalence of current users was 199 (16.5%) and out of 1195 respondents, was 169 (14.1%) at 6 months. Prevalence of tobacco use reduced significantly at 3 months *Z* = −3.1, *p* = 0.01 and at 6 months when compared with baseline *Z* = 4.2, *p* = 0.001, but not at 6 months compared with at 3 months, *Z* = 2.1, *p* = 0.09. Multivariate analysis revealed that age at initiation of tobacco use, gender, marital status, setting of dwelling, and socioeconomic status were the only variables that were associated with current tobacco use at baseline, 3 and 6 months.

**Conclusion:**

A one-time BI with a booster at 3 months had a significant effect on tobacco use in persons living in community settings. This finding suggests a need for promoting the adoption of this intervention for tobacco use in rural and semi-rural community settings.

## Introduction

While globalization of the use of cigarette and other products of tobacco is a major threat to public health worldwide (1, 2), studies have noted the decline in tobacco use in high-income countries and an increase in use in middle- and low-income countries (3, 4). For instance, Guindon and Boisclair reported that from 1970 to 2000, per capita cigarette consumption reduced by 14% in the Western world while it increased by 46% in the developing nations (3).

The growth of cigarette use in developing countries might be linked to the marketing efforts of tobacco companies, loose restrictions of tobacco control policies (5), and poor surveillance of smoking prevalence in these countries (3, 6, 7). For instance, in Nigeria, cigarette imports have increased over the years from 20 million sticks in 1970, to 198 million in 1990, and 2966 in 2000 (8). In addition, Nigeria ranks third among the largest tobacco markets in Africa after Egypt and South Africa. Despite this increase, the lifetime prevalence of tobacco use is 17% (9), and the overall prevalence of tobacco use in Nigeria is 8.9% (10), which is comparatively low compared with the US prevalence rate of 16.8% (11). Thus, Nigeria appears to be in early stages of cigarette epidemic. Estimate of deaths from smoking-attributed causes in sub-Saharan Africa reaches only 5–7% for men and 1–2% for women (12). Yet, this status and the consequences of cigarette use are not likely to respond to a quick change, partly due to weak government restrictions on tobacco use or sales.

In Nigeria, the infrastructure for tobacco control is poor despite the country’s ratification of the WHO Framework Convention on Tobacco Control (13). Nigeria has not implemented regulations such as age verification for sales, sales to minors, misleading information on packaging, the amount of tar and nicotine, product constituents as confidential information, product constituents as public information, constituent disclosure by brand, and constituent disclosure by aggregate, smoking in restaurants, nightclubs, and bars (14). Specifically, the Tobacco Control Bill, which was passed in the National Assembly about a decade ago, was yet to be adopted (15). Hence, the unbridled widespread use of tobacco in Nigeria may jeopardize future improvements in longevity that could be gained by curbing the impact of AIDS, starvation, and violence (16). Of note is the fact that attributable mortality to tobacco use is rising and if the current epidemic continues, more than 70% of these deaths are expected to occur in developing countries including Nigeria (17).

Therefore, there is a need to find other strategies that might work while efforts are ongoing to firm up tobacco restrictions at the government level in Nigeria. Screening, brief intervention, and referral for treatment (SBIRT) is a public health model that is used to screen for substance abuse and also for the delivery of low-intensity substance abuse treatments in a primary health-care setting (18). SBIRT for unhealthy drug use has been described in the scientific literature for over 50 years (19), and there has been relatively robust evidence for its effectiveness for substance use, particularly unhealthy alcohol use (20). However, some studies have noted that evidence for the effectiveness of SBIRT is much more limited for other drug use and in settings other than primary care (21).

In the Western world, the provision of SBIRT as a form of smoking cessation intervention is generally toward treatment seeking smokers (22). In developing countries such as Nigeria, where the majority live in rural settings, and access to medical care is limited to urban centers, smoking cessation treatment may not be offered until tobacco-related disease is detected or until the smoker expresses interest in quitting. Within this context, SBIRT will be particularly well suited for non-treatment seeking smokers of cigarette. In other words, while much attention has focused on primary care as a setting to promote smoking cessation, community settings could be uniquely positioned for tobacco intervention efforts in resource poor settings, such as Nigeria, where access to hospital care is limited. However, given the studies that reported inefficacy or mixed results of SBIRT, SBIRT intervention should not be taken as universally effective.

Therefore, in the developing countries, such as Nigeria, where it has not been evaluated before, there is a need for evaluative studies to guide policy and interventions. The current investigation was a single arm study designed to examine the prevalence of tobacco use and evaluate the effectiveness of SBIRT in semi-rural and rural communities. We hypothesized that participants who receive SBIRT intervention would demonstrate a decrease in cigarette consumption.

## Materials and Methods

### Study Area

Nigeria is a developing country in West Africa. Nigeria was ranked 191st among 194 member states of the World Health Organization in terms of overall health attainment. The study site was in Ibadan, Oyo State. Ibadan is the capital of Oyo state, Nigeria, and it is the third largest city in Nigeria. The city is located in the southwestern part of the country. It has a population of over 3.5 million people and 11 local government areas (23).

Ethical approval for the study was obtained from the Ethical Review Committee of the Ministry of Health, Oyo State, Nigeria. Assent was obtained from participants between 15 and 18 years and informed consent from 18 years and above.

### Study Design

A systematic stratified sampling method was used to select two local governments in Ibadan between October 2010 and April 2011. In the first stage, all 11 LGA were classified into rural or semi-rural based on government fund allocation. In the second stage, one local government was randomly chosen from each group, and in the third stage, four enumeration areas were systematically selected as clusters. The fourth stage involved the mapping and numbering of all buildings in each of the selected enumeration areas. All households within each building were serially listed in the Form specifically designed for the purpose. After getting the list of the households, simple random sampling was used to identify the households that fell within the sample. Regular households were distinguished from institutional households. All eligible respondents, who were 15 years and above in each household, were selected and were interviewed using the questionnaires including alcohol, smoking, and substance involvement screening test (ASSIST) after they gave consent/assent. The inclusion criteria for the study were both male and female tobacco users of age ≥15 years and permanent residents of study areas. The exclusion criteria were non-users of tobacco of age <15 years, not willing to get tobacco cessation intervention, and not a permanent resident of the study areas.

### Intervention Training in SBIRT and Quality Control

To increase the possibility of an effect while observing a real-world feasibility in a resource poor setting, brief interventionists were recruited from community health-care extension workers (CHEWs) in participating primary health-care clinics (*n* = 18). The CHEWs had been involved with previous surveys and agreed to adhere to the study protocol. All interventionists were trained by Victor Olufolahan Lasebikan using a group workshop followed by individual feedback on audio-taped role plays with up to five standardized patients over the course of 5 weeks (24). Treatment fidelity was assessed by scoring of 5 audio-taped role plays using Motivational Interviewing Treatment Integrity coding system (MITI 3.0) (25). Mean global scores ranged from 4.35 to 4.64 which were well above the proficiency benchmark of 4.0. All interventionists met basic motivational interviewing proficiency training goals on at least one practice role play.

A 3 days of debriefing and review of all protocols were carried out, after a pilot survey in each of the study local governments. Each interviewer had conducted two pilot interviews in the field. All questionnaires were reviewed for completeness by field coordinators. The pilot studies were carried out in a ward unit as enumerated during the National population census in each of the study local governments. This was to assess applicability of the instruments of data collection and research adherence.

### Instruments

1. A sociodemographic *pro forma* was specifically designed for this study to elicit the sociodemographic characteristics of the respondents. The questionnaire contained items such as age, age at initiation, frequency of tobacco use, marital status, socioeconomic class, and years of education. For the purpose of this study, tobacco use was synonymous with smoking.
2. The ASSIST was developed mainly to screen for drug use, but can be used for other substances, including alcohol and tobacco as well, particularly in high prevalence settings (26). The ASSIST is an eight-item instrument that screens for use of all substance types [tobacco products, alcohol, cannabis, cocaine, amphetamine-type stimulants (ATS), sedatives, hallucinogens, inhalants, opioids, and “other” drugs] and determines a risk score (“lower,” “moderate,” or “high”) for each substance (27). The risk scores are generated from item questions 2–7 for tobacco. The responses are scored from 0 to 6 based on the frequency of use of the specific substance. The risk scores are recorded on the ASSIST feedback report card which is used to give personalized feedback to clients by presenting them with the scores that they have obtained, and the associated health problems related to their level of risk. Asking clients if they are interested in viewing their scores allows the health worker to commence a discussion (brief intervention) with the client in a non-confrontational way and has been found to be a successful way of getting clients at moderate risk, in particular, to change their substance use (27). Scoring: for tobacco use, 0–3 (low risk), 4–26 (moderate risk), and 27+ (high risk). This instrument was used to score a representative sample of 1203 adolescents and adults (15 years and over) for the risk of hazardous and harmful consequences of tobacco and other substances.

The lifetime prevalence of tobacco use was obtained from Q1: “In your life, which of the following substances have you ever used (non-medical use only)?” We obtained current prevalence of tobacco use from Q2: “In the past 3 months how often have you used the substances you mentioned?” Responses were “never,” “once or twice,” “monthly,” “weekly,” and “daily/almost daily.” For the purpose of this study, use in the past 3 months was considered to be current use.

### Procedure

For those who screened positive for unhealthy tobacco use, ASSIST-Linked SBIRT was conducted as appropriate.

#### Intervention

The intervention for those who had a low risk of tobacco use (score of 0–3) was general health advice, for those with moderate risk (score of 4–26), was brief intervention and a leaflet containing information about tobacco use, and those with high risk tobacco use (score of 27+) had brief intervention, information leaflet on tobacco use and were offered referral to a specialist hospital for further assessment and treatment.

The information leaflet about tobacco use contained facts about the consequences of unhealthy tobacco use, tips for reducing the risk of tobacco-related harm, and sources of support for tobacco problems (e.g., contact details of services available in the local health district). Respondents, who had an unhealthy tobacco use, were followed up and reassessed at 3 and 6 months.

A booster brief intervention and referral to treatment was given at 3 months. Interviewers used eight anchor community members to maintain contact with members of the household, while interviewers maintained contact with these anchor persons in between interviews. Tobacco use in this study is defined as cigarette smoking.

#### Evaluation of the Intervention

The outcome of the intervention was assessed by evaluating changes in the mean number of cigarettes smoked/day at 3 and 6 months post-intervention and the mean ASSIST scores at 3 and 6 months. This is in accordance with the application of ASSIST instrument in following up clients over time. The use of mean ASSIST score has been specifically found to be highly valuable in assessing changes in ASSIST scores over time (28).

### Data Analysis

For our univariate analysis, the association between sociodemographic variables and current tobacco use was determined using the Pearson’s chi square statistics. Using the current prevalence rates at baseline, the Wilcoxon Signed-Rank test and paired *t*-test were used to determine significant changes in the proportion of tobacco users and the mean ASSIST scores at 3 and 6 months. For categorical data, all Chi squares were Yates corrected for all two levels comparisons and Bonferroni corrected for comparisons more than two levels. Following Bonferroni corrections, multiple pairwise comparisons were carried out using Chi square statistics.

Multivariate analyses were carried out using variables that were significant during univariate analysis to determine the association with tobacco use. This was carried out using binary logistic regression. To facilitate the interpretation of odds ratio, a reference category was always chosen for the independent variables with which other independent variables could be compared with tobacco use. This was done for the data at baseline, at 3 and 6 months. Analysis of data was carried out using the Statistical Program for Social Studies SPSS version 13.0.

## Results

### Enrollment and Screening

The interventionists identified a total of 1329 community dwellers as potentially eligible, of whom 1213 underwent screening. Of them, 10 were excluded because of the presence of severe general medical conditions, giving a response rate of 91.3%. The final analysis was carried out for 1203 questionnaires at baseline. At 3 months, analysis was carried out on 1199 respondents and on 1195 participants at 6 months.

### Participants’ Characteristics

The mean age of respondents at baseline was 24.45 ± 9.23 years, 51.8% were males, 66.2% were married, 47.4% had at least some secondary education, and 49.7% were of low-average socioeconomic group. Current tobacco use was more significant among males, χ^2^ = 55.2, *p* < 0.01, unmarried, χ^2^ = 9.6, *p* = 0.002, and low socioeconomic group, χ^2^ = 11.1, *p* < 0.001 (Table 1). Mean age of initiation into smoking was 17.83 (3.23) years.

**Table 1:** **Sociodemographic and other characteristics of respondents at baseline (T0) *N* = 1203**.

Variation	Total (*N* = 1203)	Current use (*n* = 248)	%	χ^2^	df	*p*
**Age (years)**						
<25	508	122	28.0	8.8	(5)	0.1
25–34	256	54	21.1			
35–44	158	25	15.8			
45–54	120	22	18.3			
55–64	111	17	15.3			
>64	50	8	16.0			
**Age at initiation (years)**						
<25	508	239	47.0	372.2		<0.001
>25	695	9	1.3			
**Gender**						
Male	623	181	29.1	55.2	(1)	<0.01
Female	580	67	11.6			
**Setting**						
Urban	487	78	16.0	10.1	(1)	<0.01
Rural	716	170	23.8			
**Marital status**						
Married	796	143	16.0	9.6	(1)	0.002
Not married	407	105	26.0			
**Education (years)**						
0	119	26	21.8	1.2	(3)	0.7
1–6	431	91	21.1			
7–12	570	111	19.5			
>12	83	20	24.1			
**Socioeconomic group^a^**						
Low	513	173	33.7	11.1	(3)	<0.001^BS^
Low average	598	68	11.4			
High average	63	6	9.5			
High	29	1	3.4			

*^a^Based on monthly wages in local currency: low – <18,500 Naira (government minimum wage), low average – 18,500–75,000 Naira; high average – 75,000–150,000 Naira, high – >150,000 Naira*.

*BS, significant following Bonferroni correction*.

### Intervention Effects

At baseline, overall lifetime prevalence and current prevalence of any tobacco products was 33.7 and 20.6%, respectively. At 3 months, prevalence of current tobacco use was 16.5% and was 14.1% at 6 months. The prevalence of tobacco use reduced significantly at 3 months *Z* = −3.1, *p* = 0.01 (RR: 0.009 < 0.04 > 0.071) and at 6 months when compared with baseline *Z* = −4.2, *p* = 0.001 (RR: 0.034 < 0.065 > 0.095), but not at 6 months compared with at 3 months, *Z* = −2.1, *p* = 0.09 (RR: −0.004 < 0.025 > 0.0536) (Table 2).

**Table 2:** **Tobacco use and associated factors: prevalence and effect of brief intervention**.

Variables	Baseline (*N* = 1203)	3 months (*N* = 1199)	6 months (*N* = 1195)	Baseline vs. 3 months	Baseline vs. 6 months	3 vs. 6 months

				Statistics *p*	Statistics *p*	Statistics *p*
Lifetime prevalence, *n* (%)	405 (33.7)	405 (33.7)	405 (33.9)			
Current use, *n* (%)	248 (20.6)	199 (16.5)	169 (14.1)	−3.1*^Z^, p* = 0.01	−4.2*^Z^, p* = 0.001	−2.1*^Z^, p* = 0.09
Male, *n* (%)	230 (19.1)	192 (16.0)	164 (13.7)			
Low risk tobacco use^a^, *n* (%)	79 (31.9)	114 (57.3)	107 (63.4)	7.4*^Z^, p* < 0.001	6.6*^Z^, p* < 0.001	1.3*^Z^, p* = 0.89
Moderate risk tobacco use^a^, *n* (%)	130 (52.4)	64 (31.7)	42 (24.9)	−8.9*^Z^, p* < 0.001	−10.1*^Z^, p* < 0.001	−3.5, *p* < 0.01
High risk tobacco use^a^, *n* (%)	39 (15.7)	21 (10.6)	20 (11.8)	−5.8*^Z^, p* = 0.001	−5.7*^Z^, p* = 0.001	1.1*^Z^, p* = 0.99
High risk tobacco + moderate or high risk alcohol use^a^, *n* (%)	36 (92.3)	16 (76.2)	13 (66.6)	−8.1*^Z^, p* < 0.001	−8.8*^Z^, p* < 0.001	1.2*^Z^, p* = 0.88
Moderate risk tobacco + moderate or high risk alcohol^a^, *n* (%)	65 (50.0)	27 (42.2)	16 (38.1)	−5.2*^Z^, p* < 0.001	−5.5*^Z^, p* < 0.001	1.3*^Z^, p* = 0.89
Mean ASSIST score (SD)	20.11 (5.56)	16.12 (3.23)	15.45 (3.1)	−5.0*^t^, p* < 0.001	−5.7*^t^, p* < 0.001	1.2*^t^, p* = 0.96
Mean (SD) daily cigarette smoking	23.76 ± 13.53	18.56 ± 10.09	17.98 ± 9.76	−7.85, *p* < 0.001	−19.8, *p* < 0.001	−0.8*^t^, p* = 0.79

*^a^Level of risk was based on ASSIST scores*.

*Z, Wilcoxon Signed-Rank Test; t, paired t-test*.

Of the current users, 79 (31.9%) scored between 0 and 3 on the ASSIST (at low health risk), 130 (52.4%) scored between 4 and 26 on the ASSIST (at moderate health risk), and 39 (15.7%) scored 27+ on the ASSIST (at high health risk). The mean ASSIST score significantly reduced at 3 and 6 months, compared with baseline measure, *t* = 5.0, *p* < 0.001 and *t* = −5.7, *p* < 0.001, respectively (Table 2).

### Referral to Treatment and Engagement

Thirty-nine (15.7%) participants had ASSIST scores ≥27 and were referred for treatment. At 3 months follow-up, 21 (10.6%) participants were referred for treatment and 20 (11.8%) at 6 months follow-up (Table 2).

Of the 39 current users at high risk of health problems, 36 (92.3%) were also at either moderate or high risk of alcohol. Sixty-five (50.0%) of the 130 current users at moderate risk of health problems were either at high or moderate risk of health problems from alcohol (Table 2).

There was a significant reduction in the mean number of cigarettes smoked per day at 3 months compared with baseline, *t* = −7.85, *p* < 0.001 and also at 6 months compared with baseline, *t* = −19.8, *p* < 0.001 (Table 2).

At 3 months, a significantly higher proportion of respondents whose age of initiation into tobacco use was <25 years were current users compared with those whose age at initiation into tobacco use was ≥25 years, χ^2^ = 309.4, *p* < 0.001. Also, a higher proportion of respondents, who were of male gender were current tobacco users compared with the female gender, χ^2^ = 200.0, *p* < 0.001. A significantly higher proportion of respondents who were rural dwellers were current tobacco users compared with the semi-rural dwellers, χ^2^ = 27.4, *p* < 0.001. A significantly higher proportion of respondents who were unmarried were current tobacco users compared with those who were married, χ^2^ = 27.1, *p* < 0.001. There was also a significant difference in the prevalence of current tobacco use across the socioeconomic status of these respondents χ^2^ = 8.01, *p* = 0.005. *Post hoc* multiple comparisons show that this difference was due to a higher current tobacco use among the low socioeconomic group, compared with the low average group χ^2^ = 28.2, *p* < 0.001, on the one hand, a higher current tobacco use among the low socioeconomic group compared with the high average group FE *p* < 0.001 and a higher current tobacco use among the low socioeconomic group compared with the high socioeconomic group FE *p* < 0.001, on the other hand.

At 6 months, a significantly higher proportion of respondents whose age at initiation into tobacco use was <25 years were current users compared with those whose age at initiation into tobacco use was ≥25 years, χ^2^ = 265.1, *p* < 0.001. Also, a higher proportion of respondents who were of male gender were current tobacco users compared with the female gender, χ^2^ = 160.0, *p* < 0.001. A significantly higher proportion of respondents who were rural dwellers were current tobacco users compared with the semi-rural dwellers, χ^2^ = 48.2, *p* < 0.001. A significantly higher proportion of respondents who were unmarried were current tobacco users compared with those who were married, χ^2^ = 52.7, *p* < 0.001. There was also a significant difference in the prevalence of current tobacco use across the socioeconomic status of these respondents χ^2^ = 8.2, *p* = 0.004. *Post hoc* multiple comparisons show that this was due to a higher current tobacco use among the low socioeconomic group compared with the low average group χ^2^ = 59.3, *p* < 0.001, on the one hand, a higher current tobacco user among the low socioeconomic group compared with the high average group FE *p* < 0.001 and a higher current tobacco user among the low socioeconomic group compared with the high socioeconomic group FE *p* < 0.001, on the other hand (Table 3).

**Table 3:** **Sociodemographic correlates of tobacco use**.

	3 months					6 months				
Variation	Total (*N* = 1199)	User (*n* = 199)	%	χ^2^	*p*	Total (*N* = 1195)	User (*n* = 169)	%	χ^2^	*p*
**Age (years)**										
<25	507	110	21.7	2.9	0.09	506	102	20.2	3.0	0.08
25–34	255	44	17.3			254	39	15.4		
35–44	157	17	10.8			156	12	7.7		
45–54	119	14	11.8			118	9	7.6		
55–64	111	10	9.0			111	5	4.5		
>64	50	4	8.0			50	2	4.0		
**Age at initiation (years)**										
<25	479	191	39.9	309.4	<0.001	477	164	34.4	265.1	<0.001
≥25	720	8	1.1			718	5	0.7		
**Gender**										
Male	604	192	29.0	200.0	<0.001	602	164	27.2	160.0	<0.001
Female	595	7	4.0			593	5	0.8		
**Setting**										
Semi-rural	479	46	9.6	27.4	<0.001	477	26	5.4	48.2	<0.001
Rural	720	153	21.2			718	143	19.9		
**Marital status**										
Married	791	99	12.5	27.1	<0.001	790	70	8.9	52.7	<0.001
Not married	408	100	24.5			405	99	24.4		
**Education (years)**										
0	118	20	20.3	1.2	0.7	116	24	20.7	0.5	0.4
1–6	431	80	18.6			431	65	15.1		
7–12	567	95	16.8			566	68	12.0		
>12	83	10	12.0			82	9	11.0		
**Socioeconomic group^a^**										
Low	511	144	28.1	8.01	0.005^BS^	511	134	26.2	8.2	0.004^BS^
Low average	596	51	8.6			596	33	5.5		
High average	62	3	4.8			62	2	3.2		
High	29	1	3.4			25	1	4.0		

*^a^Based on monthly wages in local currency: low – <18,500 Naira (government minimum wage), low average – 18,500–75,000 Naira; high average – 75,000–150,000 Naira, high – >150,000 Naira*.

*BS, significant following Bonferroni correction*.

Multivariate analysis reveals that at baseline, significant factors that remained associated with current tobacco use were age at initiation into tobacco use OR = 0.03, 95% CI (0.001–0.05), *p* < 0.001, female gender OR = 0.28, 95% CI (0.18–0.46), *p* < 0.01, being unmarried OR = 2.96, 95% CI (1.12–5.23), *p* < 0.01, high socioeconomic status OR = 0.32, 95% CI (0.19–0.59), *p* = 0.001, high average socioeconomic status, OR = 0.69, 95% CI (0.23–0.71), *p* = 0.003, low average socioeconomic status, OR = 0.65, 95% CI (0.18 = 0.78), *p* = 0.01, and being a rural dweller OR = 3.05, 95% CI (2.00–4.14), *p* = 0.001.

At 3 months, significant factors that remained associated with current tobacco use were age at initiation into tobacco use OR = 0.04, 95% CI (0.003–0.07), *p* < 0.001, female gender OR = 0.18, 95% CI (0.009–0.37), *p* < 0.001, being unmarried OR = 2.07, 95% CI (1.28–4.22), *p* < 0.01, high socioeconomic status OR = 0.31, 95% CI (0.002–0.37), *p* = 0.001, high average socioeconomic status, OR = 0.69, 95% CI (0.35–0.73), *p* = 0.002, low average socioeconomic status, OR = 0.54, 95% CI (0.17–0.61), *p* = 0.006, and being a rural dweller OR = 2.83, 95% CI (1.54–4.02), *p* = 0.002.

At 6 months, significant factors that remained associated with current tobacco use were age at initiation into tobacco use OR = 0.02, 95% CI (0.003–0.04), *p* < 0.001, female gender OR = 0.12, 95% CI (0.03–0.29), *p* < 0.001, being unmarried OR = 3.54, 95% CI (2.02–7.423), *p* = 0.001, high socioeconomic status OR = 0.32, 95% CI (0.002–0.57), *p* = 0.001, high average socioeconomic status, OR = 0.57, 95% CI (0.35–0.73), *p* = 0.002, low average socioeconomic status, OR = 0.63, 95% CI (0.49–0.81), *p* = 0.005, and being a rural dweller OR = 2.99, 95% CI (1.03–8.23), *p* < 0.001 (Table 4).

**Table 4:** **Odd ratio for current tobacco use**.

	Baseline			3 months			6 months		
Variation	OR	95% CI	*p*	OR	95% CI	*p*	OR	95% CI	*p*
**Age at initiation (years)**									
<25	1			1			1		
>25	0.03	0.001–0.05	<0.001	0.04	0.003–0.07	<0.001	0.02	0.003–0.04	<0.001
**Gender**									
Male	1			1			1		
Female	0.28	0.18–0.46	<0.01	0.18	0.009–0.37	<0.001	0.12	0.03–0.29	<0.001
**Marital status**									
Married	1			1			1		
Not married	2.96	1.12–5.23	<0.01	2.07	1.28–4.22	0.01	3.54	2.02–7.42	0.001
**Socioeconomic group**									
Low	1			1			1		
Low average	0.65	0.18–0.78	0.01	0.54	0.17–0.61	0.006	0.63	0.49–0.81	0.005
High average	0.69	0.23–0.71	0.003	0.69	0.35–0.73	0.002	0.57	0.35–0.73	0.002
High	0.32	0.19–0.59	0.001	0.31	0.002–0.37	0.001	0.32	0.002–0.57	0.001
**Setting**									
Urban	1			1			1		
Rural	3.05	2.00–4.14	0.001	2.83	1.54–4.02	0.002	2.99	1.03–8.23	<0.001

## Discussion

This study is most probably the first in sub-Saharan Africa that aimed to determine in semi-rural and rural community settings, the prevalence and correlates of tobacco use, as well as the effectiveness of ASSIST-Linked SBIRT in unhealthy tobacco users among these communities dwellers.

The lifetime prevalence of tobacco use among our participants was 33.7% and this is not much lower than the 44% lifetime use among those 15 years and older in Canada (29). We also found that the current prevalence of tobacco use was 20.6% at baseline. This is higher than the 17% prevalence reported among Nigerian adults in 2007 in a nationally representative sample (9), but is similar to the 16 and 20% prevalence of tobacco use among persons who were 15 years and above in Canada and America, respectively. It is also similar to the overall tobacco use prevalence of 21% in India (30). However, compared with our estimates, the 2012 Global Adult tobacco Survey (GATS) that was conducted in 16 countries found higher current tobacco use prevalence in North America, Europe, and South Asia (31). Nonetheless, our finding suggests that tobacco use might be assuming epidemic proportions in people who live in rural and semi-rural settings in Nigeria.

Another key finding in our study is the group of correlates of tobacco use among current users. Those who used tobacco were more likely to have started around 17 years of age, to be males, unmarried, of low socioeconomic status and live in a rural setting. With respect to the age at initiation of tobacco use, this is similar to the age at smoking initiation that is before the age of 18 years in Western countries (32). We acknowledge that the average age at tobacco initiation varies by country, income, education, and age cohort (31, 33–35). In addition, we note that differences in age at initiation by country may reflect variation in stages of tobacco use epidemic between countries or the complexity of tobacco control measures implemented (36). We cautioned above that tobacco use in the rural and semi-rural settings in Nigeria might be assuming epidemic proportions. In line with this, we situate our current finding of lower age at initiation of tobacco use as highlighting a significant problem that could be faced in the future of the health consequences of tobacco use and dependency on tobacco.

From the foregoing, one could justifiably ask “what could make youths especially in rural settings use tobacco at an earlier age?” Our data deductively serve to guide and stimulate additional research. In addition, priority needs to be given to the development of country specific tobacco control programs for adolescents and youths. Furthermore, given the public health importance of tobacco-related diseases such as CVD and other CVD risk factors (e.g., diabetes, hypertension) (37, 38), it is critical to track tobacco use within specific contexts (i.e., rural vs. semi-rural settings) in Nigeria and to characterize tobacco use patterns in terms of populations that could be vulnerable to tobacco use.

Concerning other correlates, our study aligns with prior research (30, 38–40). In both India and Pakistan, some predictors of tobacco use are male gender, low socioeconomic status, and rural geographic location (30, 41). We confirm that male gender is a correlate of tobacco use in low-income countries, in contrast to middle- and high-income countries where the male preponderance is blurring (42). Similar to the male gender contrast, our findings as regards the association between tobacco use and rural dwelling is in line with low-income countries (43) but dissimilar to findings in middle- and high-income countries where tobacco use correlates with dwellers in large cities (44). However, our result with respect to a positive relationship between tobacco use and low socioeconomic status is in agreement with Western findings (44) as well as findings from other developing nations (30, 38, 39). It is possible that unawareness of the health risks of tobacco use might be the factor that ties these associations together. Further research is needed to tease out the possible moderating influence of lack of information on the health risk of tobacco use.

Our observation of an associated alcohol-related health risk among respondents with tobacco-related health risks is illustrative of the co-use of both tobacco and alcohol. Studies have found that smokers are much more likely to use alcohol and vice versa (45). Also, tobacco and alcohol share a similar psychological mechanism as subjective mood altering chemicals that are socially learned and are used by some individuals as coping mechanisms (46). Moreover, repeated use acts as positive reinforcement (47); the pharmacological dependence of nicotine usually increases the probability of alcohol use, usually in social settings because of the social acceptability of alcohol.

A major finding in this study is that it underscores the usefulness and applicability of SBIRT in the hands of CHEW and the positive impact of SBIRT delivered through CHEW on tobacco use as well as unhealthy use in a semi-rural community setting. This current study is important in three ways: (1) it focused on tobacco, and not alcohol, (2) SBIRT was deliverable by CHEW rather than by clinicians only, and (3) it enrolled people in the community with poor access to orthodox medicine and who might not seek treatment rather than those who went to the hospital or were admitted in emergency settings.

In rural and semi-rural community settings, we investigated the usefulness of a single session of brief intervention with a booster session in reducing tobacco use. A major finding in this assessment was that the rate of tobacco use reduced significantly between baseline and 3 and 6 months, respectively. There were significant shifts from high risk to moderate and low risk use of tobacco.

Our study was limited by a number of factors. First, we did not stratify the users into different stages of change. In other words, we could not assess the impact of different stages of change in unhealthy tobacco use in our study population. This is very relevant considering reports indicating that psychosocial interventions, that target behavioral change often do not yield a significant effect (48). We therefore recommend further studies to explore the possible influence of stages of change in tobacco use reduction. Second, we did not assess tobacco cessation and tobacco cessation in relation to SBIRT. The main objective of SBIRT being cessation from substance use. Future studies are required to access tobacco cessation following SBIRT, because the reduction in the tobacco use rate does not equate cessation. Third, we did not include a control group. This has greatly limited the interpretation of the effect of the intervention. Fourth, all our analysis was based on self-reports. Future works require, including a toxicological screen to their methodology. Fifth, we also did not allocate any diagnosis to the tobacco users; therefore, it was difficult to determine if the effect of the intervention was on sparing users or long-term users.

In conclusion, while tobacco use is reaching epidemic proportions in rural and semi-rural settings in Nigeria and is associated with male gender, early age at initiation into use, low socioeconomic status, and living in rural areas, SBIRT promises to have implementation potentials in delivering the intervention for the reduction of tobacco use and unhealthy tobacco use in semi-rural community in Nigeria.

## Author Contributions

VL conceived the idea and was responsible for study design, analysis, and manuscript writing. BO was responsible for data collection and was also involved in manuscript writing. Both authors gave a substantial contribution to the study.

## Conflict of Interest Statement

The authors declare that the research was conducted in the absence of any commercial or financial relationships that could be construed as a potential conflict of interest.
